# Solid‐State Nuclear Magnetic Resonance (SSNMR) Characterization of Osteoblasts From Mesenchymal Stromal Cell Differentiation to Osteoblast Mineralization

**DOI:** 10.1002/jbm4.10662

**Published:** 2022-09-12

**Authors:** Jing‐Yu Lin, Ming‐Hui Sun, Jing Zhang, Meng Hu, Yu‐Teng Zeng, Qian‐Qian Yi, Jian Wang, Yun Bai, Yifeng Zhang, Jun‐Xia Lu

**Affiliations:** ^1^ School of Life Science and Technology ShanghaiTech University Shanghai China; ^2^ University of Chinese Academy of Sciences Beijing China; ^3^ Shanghai Clinical Research and Trial Center ShanghaiTech University Shanghai China

**Keywords:** MESENCHYMAL STROMAL CELLS, OSTEOBLAST, SOLID‐STATE NMR, EXTRACELLULAR MATRIX, MINERALIZATION

## Abstract

Solid‐state nuclear magnetic resonance (SSNMR), a technique capable of studying solid or semisolid biological samples, was first applied to study the cell differentiation and mineralization using the whole‐cell sample. Mesenchymal stromal cells (MSCs) with multipotent differentiation capacity were induced to differentiate into osteoblasts. The whole differentiation process, osteoblast mineralization and the mineral maturation, was investigated using SSNMR, providing intact, atomic level information on the cellular mineral structural transformation. Our research indicated the extent of osteoblast mineralization could vary significantly for different cell populations whereas the difference was not easily shown by other means of characterization. The SSNMR spectra revealed hydroxylapatite (or hydroxyapatite [HAP]) formation around 2 to 4 weeks after osteogenic induction for MSCs with a high differentiation potency. The early mineral phase deposit before HAP formation contained a high amount of HPO_4_^2−^. The structures of minerals in the extracellular matrix (ECM) of osteoblasts could evolve for a period of time, even after the incubation of cells has been stopped. This observation was only possible by studying the sample in an intact state, where ECM was not disturbed. These findings improved our understanding of MSCs, which had wide applications in bone regeneration and tissue engineering. Meanwhile, this work demonstrated the advantage of studying these cellular systems as a whole without any mineral extraction, which had been largely overlooked. © 2022 The Authors. *JBMR Plus* published by Wiley Periodicals LLC on behalf of American Society for Bone and Mineral Research.

## Graphical abstract

The figure describes the three stages of mineralization. First mesenchymal stromal cells are induced to differentiate into osteoblasts and later osteoblast mineralization would finally result into mineral maturation and bone formation. The whole process at these three stages is carefully characterized by SSNMR in this research.

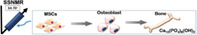

## Introduction

In bone development, cells develop into various stages, and the cell differentiation and apoptosis are highly regulated. In vitro, mesenchymal stromal cells (MSCs), also referred as mesenchymal stem cells, are widely applied in bone regeneration and tissue engineering as a tool to culture artificial bone materials. Osteogenic differentiation of MSCs into osteoblasts could be induced by a combination of chemicals with β‐glycerophosphate (β‐GPO_4_), ascorbic acid (Asc), and dexamethasone (Dex) in vitro.^(^ ^1^ ^)^ Examination of the cellular processes has been carried out using morphological evaluations, immunohistochemical methods, and biochemical methods.^(^ ^2^, ^3^ ^)^ However, the morphological examination could not provide the molecular detail information for a clear characterization of the cellular mineralization status. The MSCs differentiation into osteoblasts is also marked by a upregulation of alkaline phosphatase activity, deposition of the mineralized extracellular matrix (ECM), and secretion of sialoprotein, osteopontin, etc.^(^ ^4^ ^)^ However, how these molecular markers are related to bone quality and the hydroxylapatite (also hydroxyapatite [HAP]) formation are still not clear. To characterize the cell differentiation and its mineralization is the first step for us in designing artificial bone material and would also provide us useful information in understanding bone development in vivo. The techniques utilized that are capable of providing the mineral structural information at the molecular level include Raman spectroscopy, Fourier transform‐infrared spectroscopy (FTIR), X‐ray diffraction (XRD), nuclear magnetic resonance (NMR), etc.

However, when studying the calcium phosphate crystals deposited in osteoblast cell cultures, it has been common practice to remove the organic matrix components and cells from the mineral to facilitate the mineral structure studies.^(^ ^5^, ^6^, ^7^ ^)^ Those steps may disturb the original state of the system, preventing an accurate characterization. These mineral extraction procedures also prevented us from studying the protein/mineral interaction or gaining the correlation between the cellular status and the mineral structure. Solid‐state nuclear magnetic resonance (SSNMR) is a powerful technique for the study of non‐crystalline, insoluble samples. It has been used to extensively study bone and mineral structures. SSNMR could potentially provide a way to study the whole cell directly and noninvasively without purification of the specific component. In this work, we tested this hypothesis and demonstrated that SSNMR spectra were sensitive to the changes of the cell during its differentiation and mineralization, revealing definite markers for osteogenic differentiation. The studies on the whole cell sample also provided the intact information on a direct response of cells to chemical treatments at the molecular level.

Studying the whole cell using nuclear magnetic resonance (NMR) has been limited in the literature.^(^ ^8^, ^9^, ^10^, ^11^ ^)^ Because of the complexity and heterogeneous nature of the sample, the NMR studies are usually hindered by low resolution and sensitivity.^(^ ^9^, ^12^ ^)^ Virus particles, especially the bacteriophages, have been studied extensively and successfully, benefiting from the fact that the phage structure is dominated by a single protein with a relatively uniform conformation.^(^ ^13^, ^14^, ^15^ ^)^ In this research, phosphorus (^31^P) nuclei were mainly utilized to trace the structural changes of the formed minerals during the cells’ differentiation with high sensitivity and good resolution. The correlation between the collagen in ECM and HAP formation was observed. The rigid environment provided by ECM and the mineral also facilitated the SSNMR studies.

## Materials and Methods

### Cell culture

All animal procedures were conducted following the Ethics Committee and approved by the Administrative Panel on Laboratory Animal Care (protocol no. 20210914001) of ShanghaiTech University. Bone marrow MSCs were harvested from the femurs and tibias of SD (Sprague‐Dawley) rats using the method described^(^ ^16^ ^)^ after the rats were euthanized by tribromoethanol (Avertin). Total three rats were used for three independent cell induction studies, one was a 3‐month‐old SD rat and the other two were both 2‐week‐old SD rats. Briefly, after the femurs and tibias were carefully washed, the bone marrow was flushed using α minimum essential medium (α‐MEM; Meilunbio, Dalian, China). Following 48 hours of incubation under a standard culture environment in α‐MEM containing 10% fetal bovine serum (FBS; Gibco, Grand Island, NY, USA) and 1%Gibco Penicillin‐Streptomycin (P/S; Life technologies, Carlsbad, USA), the adherent cell population was left and grown in culture dishes. The cells were further detached and purified, and the cells in passage 3 were used in the following experiments.

Cells (100,000 cells/mL) were seeded in 15‐cm culture dishes for NMR. The osteogenic‐inducing medium was composed of Dulbecco's Modified Eagle Medium (DMEM; Meilunbio, Dalian,China), 10% FBS, 1% P/S, 200μM dexamethasone (Dex; Solarbio, Beijing, China), 20μM β‐glycerophosphate (β‐GPO_4_; Meilunbio,Dalian, China), and 50μM (or 400μM) l‐ascorbic acid (Asc; Solarbio, Beijing, China). The culture medium of each group was replaced with a fresh inducing medium every 3 days. The cells were induced for different periods. Before collecting the cell for NMR, the cell plates were washed three times with PBS. Cells were collected by trypsin(trypsin; Meilunbio, Dalian, China) digestion method to release them from the culture dishes. Additional washing was carried out by pipetting PBS many times on the bottom of the plates to make sure that the cells and the ECM were released from the plates and collected for NMR studies. For the first cell induction, the cells were extracted from a 3‐month‐old SD rat and only 50μM Asc was supplemented to the DMEM medium. For the second and third preparation, the cells were extracted from 2‐week‐old SD rats and 400μM Asc was supplemented.

In parallel, cells (100,000 cells/mL) were seeded in 96‐well plates for Alizarin Red S staining (ARS; Solarbio, Beijing, China). The osteogenic‐inducing medium was the same as the 15‐cm culture. The culture medium of each group was replaced with a fresh inducing medium every 3 days. After cells being induced for 0, 7, 14, and 21 days, the plates were washed and then fixed for 10 minutes and stained by 0.2% ARS (Solarbio) staining solution (pH = 4.2) for 30 minutes at 37°C. The ARS was carried out only for the second preparation.

### Sample preparation for SSNMR spectroscopy

The whole‐cell culture from a 15‐cm dish was collected by centrifugation at 800*g*, 24°C for 2 minutes for each NMR sample. After the supernatant PBS was removed, the cell sample was lyophilized for 3 hours or overnight. The wet cell sample was collected by removing the supernatant PBS first and then the pellet was directly transferred to a 3.2‐mm SSNMR rotor by centrifugation at 1520*g*, 24°C for 5 minutes. For the bone sample, after removing the attached muscle, the whole lower limb bone was ground to powder and lyophilized overnight. The dry mass of the cell sample varied from 2 to 12 mg for different preparations and the weight of the bone powder sample was 31.6 mg.

### Magic angle spinning SSNMR studies

Magic angle spinning (MAS) SSNMR experiments were acquired on a 16.4‐T (700 MHz ^1^H frequency) Bruker AVANCE NEO spectrometer with a 3.2‐mm triple‐resonance HCP MAS probe. The temperature for all experiments was set at 288 K. ^13^C chemical shifts were externally referenced to sodium trimethylsilylpropanesulfonate (DSS) by setting a downfield ^13^C signal of adamantine to 40.48 part per million (ppm).^(^ ^17^ ^)^ The ^31^P chemical shifts were indirectly referenced to 85% H_3_PO_4_ (0 ppm) using the ^31^P/^1^H resonance frequency ratio 0.404807420. All of the experiments were collected with a 2‐second recycle delay.

For ^1^H one pulse experiment, ^1^H 90‐degree pulse was 3.1 μs. For ^1^H‐^13^C cross‐polarization (CP) MAS^(^ ^18^, ^19^ ^)^ match at 15 kHz MAS, the ^13^C radiofrequency (rf) strength was set to 50.9 kHz, ^1^H rf strength was set to 79.8 kHz. The CP contact time was 200 μs, optimized for the protein Cα. For ^1^H‐^31^P CP match at 15 kHz MAS, the ^31^P rf strength was set to 48.8 kHz, ^1^H rf strength was set to 66.5 kHz. The contact time was 1.5 ms. For ^1^H‐^31^P CP match at 0 kHz MAS, the ^31^P rf strength was set to 39.0 kHz, ^1^H rf strength was set to 38.0 kHz. The contact time was 1.5 ms. ^1^H Small Phase Incremental ALteration (SPINAL‐64) (individual pulse width = 6.2 μs) decoupling was applied during the evolution and acquisition periods.^(^ ^20^ ^)^

For two‐dimensional (2D) ^1^H‐^31^P heteronuclear correlation (HETCOR), NMR spectra were acquired using a ^1^H 3.2‐μs pulse width (90 degree flip angle) corresponding to an rf of 77.9 kHz.^(^ ^21^ ^)^ The cross‐polarization step was performed using a contact time of 1.5 ms with a 70%–100% ramp shape of 75.4 kHz on the ^1^H channel and a square‐shaped pulse of 52.3 kHz on the ^31^P channel, a spinning rate of 15 kHz. A SPINAL‐64 decoupling scheme was used with pulse length of 6.2 μs at an rf field strength of 77.9 kHz.^(^ ^22^ ^)^

For collagen‐type I sample (Solarbio; CAS:9007‐34‐5, from bovine Achilles tendon), the spectrum was acquired on a 3.2‐mm triple‐resonance HCN MAS probe. The temperature was set at 288 K. For ^1^H‐^13^C CP MAS^(^ ^18^, ^19^ ^)^ match at 15 kHz MAS, the ^13^C rf strength was set to 74.63 kHz, ^1^H rf strength was set to 87.72 kHz. The CP contact time was 200 μs, optimized for the protein Cα.

### XRD method

The MSC induction products and a 4‐week‐old rat bone (femur and tibia) sample for NMR measurement were also tested using XRD. XRD of the powder samples were recorded on a D8 VENTURE X‐ray Diffractometer (Bruker, Karlsruhe, Germany) using Cu Kα radiation (λ = 1.54184 Å) with an acceleration voltage of 50 kV and a current of 1 mA at 298 K or 150 K. The distance from the crystal to the detector was 50 mm with 60‐second exposure time.^(^ ^23^ ^)^

### Transmission electron microscopy

The transmission electron microscopy (TEM) images were acquired by a Tecnai G2 Spirit Transmission Electron Microscope (Thermo Fisher Scientific, Waltham, MA, USA) with an acceleration voltage of 120 kV. All the cell samples were first resuspended using 20 μL double‐distilled water (ddH_2_O) and then centrifuged at 800*g*, 24°C for 10 seconds. Five microliters (5 μL) of supernatant was applied to coat the carbon‐coated copper grid (300 meshes; Beijing Zhongjingkeyi Technology Co., Ltd., Beijing, China) for 60 seconds. The grid was washed twice using 5 μL ddH_2_O for 45 seconds then 5 μL of 2% uranyl acetate was applied for 60 seconds. In each step, the solution was blotted away using filter paper touching the edges of the grids.

## Results

### MSCs from different rats and with different chemical treatments displayed different mineralization ability revealed by SSNMR

First, MSCs from the femur and tibia bone marrow of a 3‐month‐old and a 2‐week‐old SD rat were extracted according to the literature.^(^ ^16^ ^)^ The cells were incubated using DMEM supplemented with 10% FBS and 1% P/S. The adherent cell population growing in colonies was selected for further experiments. Osteogenic differentiation was induced by treating the cells with a combination of Dex, β‐GPO_4_, and Asc for 14 days. Two preparations were carried out using 200nM Dex, 20mM β‐GPO_4_ but two different concentrations of Asc (50μM for MSCs from the 3‐month‐old rat, 400μM for MSCs from the 2‐week‐old rat). It has been demonstrated that Dex induces MSC differentiation into osteoblasts by activating osteogenic transcription factor *Runx2* expression. β‐GPO_4_ could not only provide the cells a phosphate source, but also act as a signaling molecule to regulate the expression of many osteogenic genes in the cells. Asc is a cofactor for enzymes that hydroxylate proline and lysine in pro‐collagen, thereby facilitating the secretion of collagen type I in the ECM and subsequently leading to the gene expression of osteogenic proteins in the nucleus.^(^ ^1^ ^)^

**Fig. 1:**
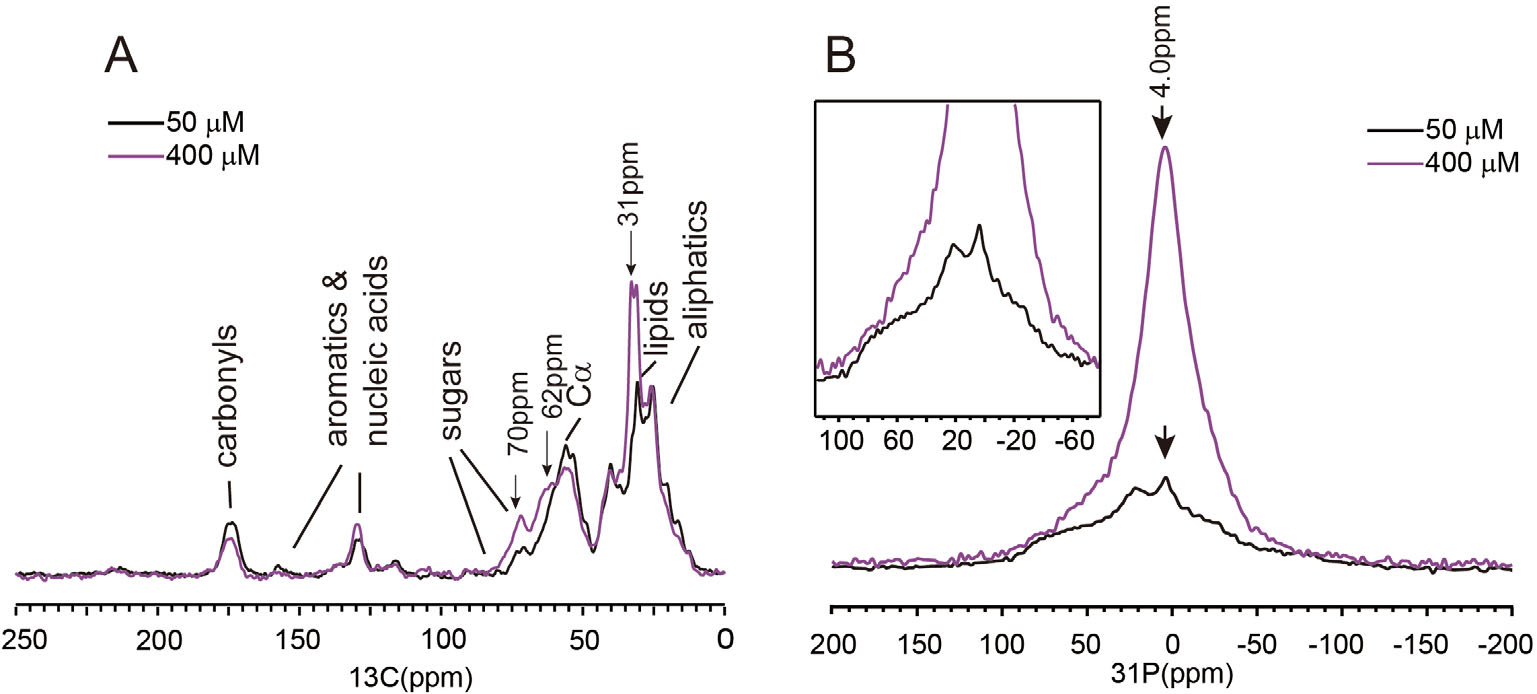
Comparison of SSNMR spectra of MSCs osteogenically induced by a cocktail of 200nM Dex, 20mM β‐GPO_4_ and Asc (50μM shown in black, total sample weight of 11.9 mg and 400μM shown in purple, total sample weight of 4.7 mg) for 14 days. Black arrows indicated peaks where big differences were shown. (*A*) ^1^H‐^13^C CP spectra of the whole cell samples, measured at 15 kHz MAS. A total of 17,141 scans were obtained for the sample using 50μM Asc induction, and 23,715 scans were collected for 400μM Asc induction. (*B*) Static ^1^H‐^31^P CP spectra of the whole cell samples. Inset is an expanded view. A total of 10,000 scans were collected for the cell sample using 50μM Asc induction, whereas 2048 scans were collected for the cell sample using 400μM Asc induction.

Figure 1 shows the SSNMR spectra of the two samples at 10°C. The entire whole‐cell culture in one 15‐cm dish was collected and transferred to the NMR sample tube for study. The NMR spectra in this work were collected on the freeze‐dried samples unless otherwise stated. The dried condition was used to maintain the stability of the cell samples. ^13^C CP spectra in Fig. 1*A* displayed typical ^13^C spectra of the cells. The assignment of the ^13^C spectrum for 50μM Asc treatment of MSCs was indicated in the figure as a general guidance. The assignment was taken from the literature report for HeLa, CHO, and HEK whole cells because the spectrum was very similar to the report.^(^ ^12^ ^)^ Slightly different, stronger peaks around 70 ppm, 62 ppm, and 31 ppm were apparent for the high concentration of Asc treatment of the cells. The peak positions were close to the hydroxyproline Cγ (~70 ppm), proline Cα hydroxyproline Cα or Cδ (~60 ppm), proline Cβ (~30 ppm) carbon positions of collagen^(^ ^24^ ^)^ (The assignment of ^13^C spectrum of collagen is shown in Fig. 5*C*.). Although the resolution of one‐dimensional (1D) ^13^C spectra was low, the result suggested a high collagen content for the cells treated with 400μM Asc. The peak at 31 ppm may also suggest a higher amount of lipids in the sample.

Static ^1^H‐^31^P CP spectra (Fig. 1*B*) showed bigger differences in the line shape. The sample with 50μM Asc treatment showed very broad ^31^P chemical shift anisotropic (CSA) patterns with the width from ~100 ppm to ~ −100 ppm. This line shape was consistent with the contribution from both the broader ^31^P CSA of the rigid DNA and a relatively narrower ^31^P CSA of membrane lipids, as suggested in the literature using the *Escherichia coli* (*E. coli*) cells.^(^ ^25^ ^)^ The ^31^P CSA of rigid DNA in the cell could reach ±100 ppm.^(^ ^9^ ^)^ The deconvolution of the spectra using DMFIT (A software developed by Dominque Massiot at CEMHTI UPR3079 of CNRS, Orléan, France)^(^ ^26^ ^)^ with two CSA components was shown in Fig. S1. Both spectra exhibited a similar width (Fig.1*B*, inset), which was determined by the broader ^31^P CSA of the rigid DNA. The sample with the high concentration of Asc treatment showed a dominant central peak around 4 ppm. Therefore, the deconvolution of the spectra in Fig. S1 also included one isotropic component. The stronger central peak (component 3, the isotropic component in the deconvolution) increased significantly for MSCs treated with 400μM Asc, consistent with a higher amount of the phosphate mineral deposit formed in the cells’ ECM. The ^31^P peak of the mineral was not isotropic in reality. For HAP, the ^31^P CSA width (defined by $\left|\sigma_{33}-\sigma_{11}\right|$) was reported to be about 40 ppm.^(^ ^27^ ^)^ However, because the CSA width of minerals is much smaller than that of DNA, for simplification, a broad isotropic peak (~30 ppm line width at the half height) was used for the 400μM Asc condition. The experiment result confirmed that the cell sample with a high concentration of Asc of 400μM had a higher osteogenic differentiation ability to osteoblast and thereby a higher mineral content. The overall ^31^P signal area was compared for the two conditions, after correcting for differences in sample masses and numbers of scans. It showed that the sample treated with 400μM Asc had a much stronger signal for the minerals (~200 times) and three to four times the signals for the organic phosphorus. Because the MSCs treated with 50μM Asc was also from an older rat, both the age of the rat and the concentration of Asc could be the cause for the different differentiation and mineralization ability.

### SSNMR was sensitive to the changes of cells during the differentiation

**Fig. 2:**
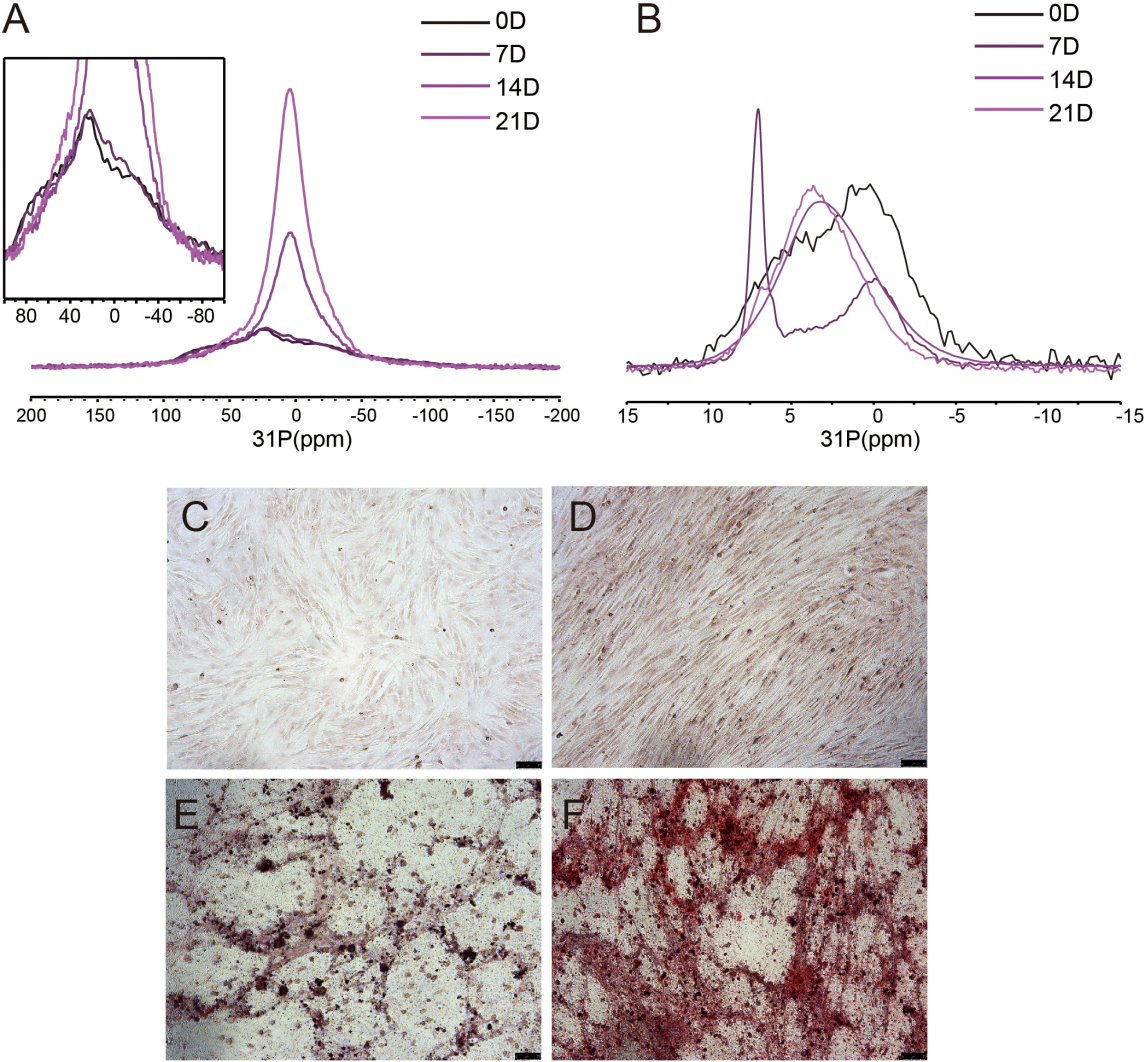
(*A*,*B*) SSNMR spectra of cells obtained from the second preparation of osteogenic induction of MSCs for a period of 21 days using 200nM Dex, 20mM β‐GPO_4_, and 400μM Asc. (*A*) Static ^1^H‐^31^P CP spectra for MSCs. (*B*) ^1^H‐^31^P CP spectra for MSCs measured at 15 kHz MAS. The sample weight was 5.8 mg, 6.1 mg, 4.7 mg, and 2.5 mg for 0, 7, 14, and 21 days of induction, respectively. For *A*, 31,665 scans were collected for 0 days and 7 days of induction, but only 2048 scans for 14 days and 21 days induction. For *B*, 1469 scans were obtained for 0 days of induction, 10,000 scans, 4000 scans, and 128 scans were obtained for 7 days, 14 days, and 21 days of induction. (*C*–*F*) Optical microscopic observation of Alizarin red staining of osteogenically induced MSCs using 200nM Dex, 20mM β‐GPO4, and 400μM Asc for 0, 7, 14, and 21 days, respectively. Scale bars = 75 μm.

The change of MSC status at the molecular level before induction and after osteogenic induction for a period of 21 days was also monitored for the MSCs from a 2‐week‐old rat using ^31^P CP SSNMR (Fig. 2*A*,*B*). The osteogenic induction was carried out with 400μM Asc in addition to 200nM Dex and 20mM β‐GPO_4_. Significant changes of the ^31^P CP spectra were observed both at the static condition and at 15 kHz MAS. Starting from the 14th day, the static ^31^P NMR spectra were dominated by the central peak from the mineral deposit (Fig. 2*A*). The isotropic peak region of ^31^P CP spectra at 15 kHz MAS is also displayed in Fig. 2*B*, with the full spectra in Fig. S2. The isotropic peaks were displayed as two merged broad peaks on day 0, two well‐separated peaks on day 7, and one broad peak on the 14th and 21st days. Using DMFIT,^(^ ^26^ ^)^ the full spectra were tentatively deconvoluted into two major ^31^P components: one has an isotropic peak positioned at around 4–7 ppm with a narrower CSA and the other has an isotropic peak positioned at around 0–1 ppm with a broader CSA. With the increase of the cell induction time, the contribution of the narrower CSA component increased, whereas the contribution of the broader CSA component decreased (Fig. S2). The increase of the narrower component reflected the increase of the mineral deposit in ECM. The broader component has a δ_CS_ (δ_CS_ = σ_33_‐σ_iso_) value varied around −100 ppm (from −117 ppm to −84 ppm), consistent with a contribution mainly from DNA. However, it was hard to relate each component in the spectrum exactly to a specific ^31^P species in the cell, and each component may also be contributed by a combination of different ^31^P species in the cell.

Alizarin red S (ARS) specifically labels the calcium ions in the mineral deposit. Figure 2*C*–*F* showed the cell morphology changes during the 21 days using ARS staining observed by the optical microscope. On day 7, the cells became more aligned and tightly packed to each other. And a large number of mineral clusters shown as red dots could be observed on the 14th and 21st days. Particles could also be observed using an optical microscope in the culture dishes without staining (the microscopic image for the sample on the 21st day is shown in Fig. S3*A*). With the cells' differentiation into osteoblasts and more mineral forming in the ECM in the period of 21 days, the cell environment and dynamics were expected to change significantly. Therefore, the ^31^P spectra CSA patterns contributing from specific species were also expected to change. It was reported that the increased interactions between the cells and the collagen in the ECM played a key role for the maintenance of osteoblast differentiation.^(^ ^28^, ^29^ ^)^

**Fig. 3:**
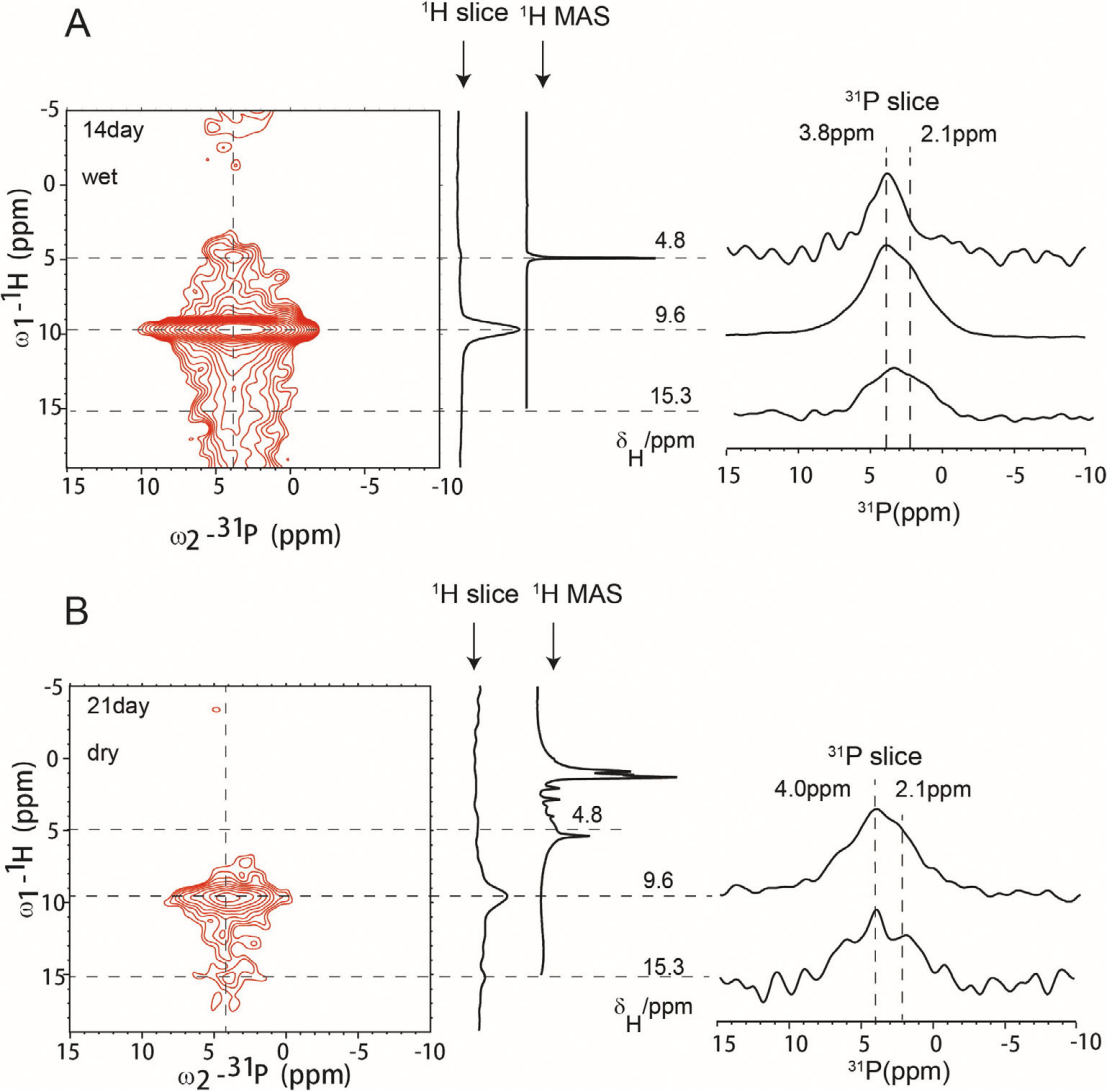
^1^H–^31^P 2D HETCOR spectra of MSCs. (*A*) MSCs after osteogenic induction for 14 days. MSC sample was directly collected from centrifugation without further drying (labeled as “wet”) and (*B*) MSCs after osteogenic induction for 21 days and the cells were freeze‐dried before NMR studies (“dry”). Both spectra were collected from 128 scans.

It has been believed that the mineral formed in the ECM of osteoblast contained HAP (Ca_5_(PO_4_)_3_OH), the main mineral component in the bone. However, neither the positive staining result of ARS nor the mineral particles under microscope was enough for a confirmative conclusion. 2D ^1^H‐^31^P HETCOR experiments were carried out to gain the mineral identity in our samples. HAP has a very characteristic 2D ^1^H‐^31^P HETCOR spectrum, showing a correlation peak corresponding to the hydroxyl group interaction with ^31^P at (^1^H/^31^P ~ 0 ppm/~3.8 ppm).^(^ ^30^ ^)^ Figure 3 displays the HETCOR spectra for the cell samples obtained on the 14th day and 21st day of the induction. The sample on the 14th day was a wet cell sample directly from centrifugation without drying. The ^1^H ^1^D MAS spectrum showed the dominant water peaks at 4.8 ppm and no other ^1^H peaks could be observed because of the abundance of water (Fig. 3*A*, ^1^H MAS). In the 2D ^1^H‐^31^P HETCOR spectrum, a weak peak was observed at ^1^H 4.8 ppm, but a big correlation peak was displayed at ^1^H 9.6 ppm, ^31^P 3.8 ppm. The water‐^31^P correlation peak indicated that although the water ^1^H is dominant in the proton 1D spectrum, very few water protons have strong interactions with ^31^P nuclei. It was from small amounts of water molecules tight bound to the minerals. The big correlation peak with ^1^H chemical shift close to 10 ppm, indicated an acidic proton from HPO_4_^2−^.^(^ ^31^ ^)^ The 2D spectra also displayed broad and weak signals at ^1^H ~ 15 ppm, suggesting trace amounts of H_2_PO_4_^−^, whose protons were involved in stronger hydrogen bonding interactions. The sample on the 21st day was freeze‐dried. Many ^1^H peaks at the 0–5 ppm region were visible in the 1D ^1^H MAS spectrum because the big water peak was removed (Fig. 3*B*, ^1^H MAS). The 2D ^1^H‐^31^P HETCOR spectrum indicated a correlation peak at ^1^H 9.6 ppm, ^31^P 4.0 ppm with a minor peak at ^1^H 15.3 ppm, ^31^P 4.0 ppm, consistent with the forming of minerals containing HPO_4_^2−^ and H_2_PO_4_^−^. Different from the wet sample on the 14th day, the correlation signal between the mineral and the surface‐bound water disappeared upon drying on the 21st day sample. Almost all ^31^P 1D slices showed a shoulder at ^1^H 2.1 ppm, although the ^31^P 1D slice at ^1^H 4.8 ppm was centered at 3.8 ppm, with almost no intensity at 2.1 ppm. The broad peaks indicated the heterogeneity of the minerals. Interestingly, no correlation peak was observed at ^1^H/^31^P (~0 ppm/~3.8 ppm), suggesting HAP was not formed yet for the cells after 21 days of induction in this preparation (we called it the second preparation).

### MSCs from rats with the same age could be very different in their differentiation ability

**Fig. 4:**
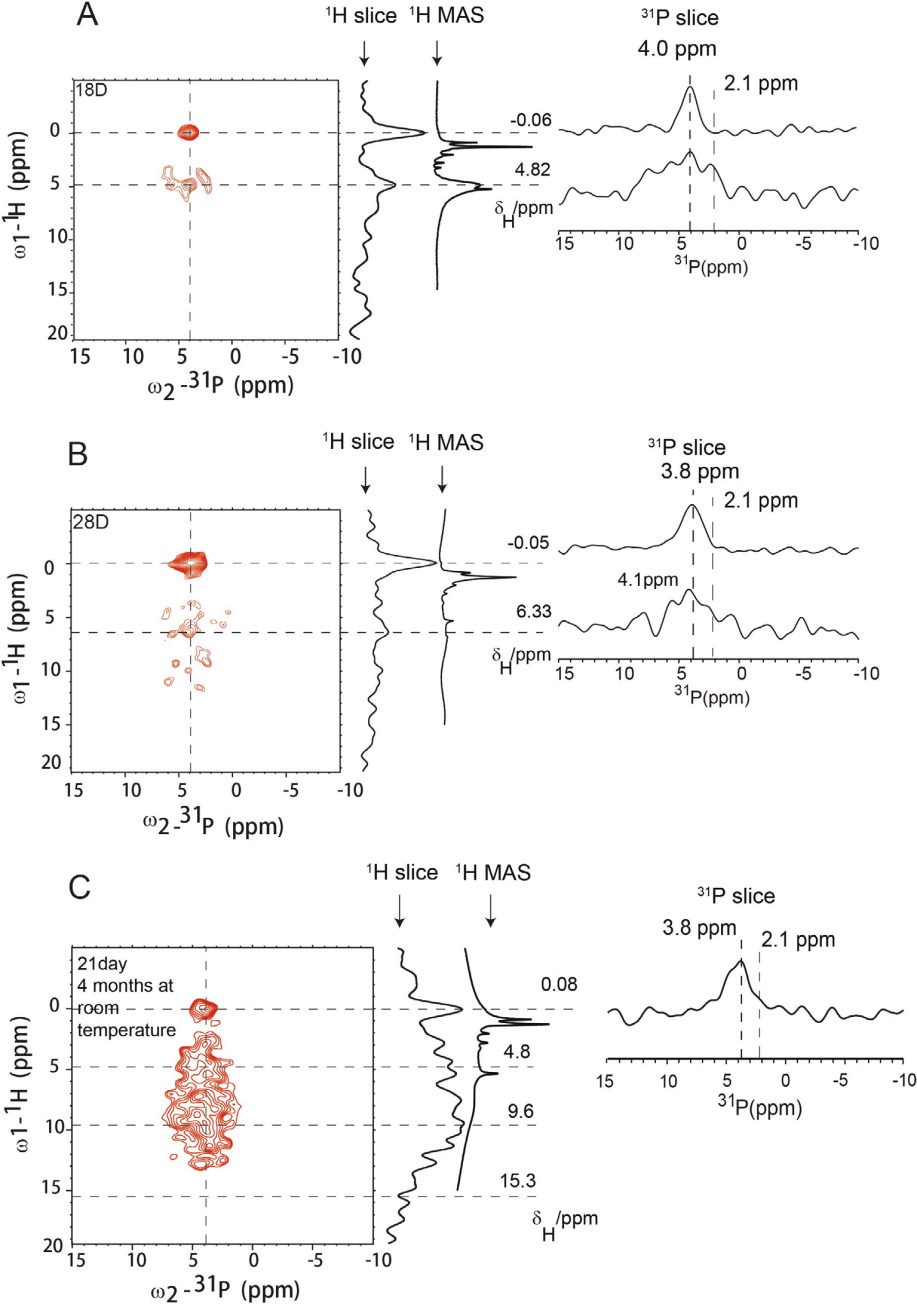
^1^H‐^31^P 2D HETCOR spectra of MSCs. (*A*,*B*) For the third preparation, where the spectra of MSCs were collected after osteogenic induction for 18 days and 28 days, respectively. The cells were freeze‐dried before NMR studies. (*C*) The sample for Fig. 3*B* was left at room temperature for more than 4 months. All spectra were averaged from 70 scans.

In order to gain a better understanding of the osteoblast cells mineralization in vitro, a third preparation was carried out. The second SD rat of 2 weeks old was euthanized and MSCs from the femur and tibia bone marrow were extracted. A new round of osteogenic induction of MSCs was carried out using the same conditions as the second preparation. The optical microscopic image of the cells after 28 days of induction is shown in Fig. S3*B* with dark spots, suggesting the mineral formation. The 1D ^31^P CP spectra and 2D ^1^H–^31^P HETCOR at 15 kHz MAS are shown in Figs. S4 and 4, respectively. To our surprise, the 2D ^1^H–^31^P HETCOR spectra of MSCs after 18 days (Fig. 4*A*) and 28 days (Fig. 4*B*) of osteogenic induction both displayed a strong correlation peak at ^1^H/^31^P (~0.0 ppm/~4.0 ppm, the OH^−^/PO_4_^3−^ correlation) with a narrow linewidth (exhibited by the low intensity of ^31^P 1D slices at 2.1 ppm), indicating the formation of HAP. Both samples were freeze‐dried, therefore no strong water correlation peak at ^1^H 4.8 ppm position. Figure 4*A* shows a small water/^31^P correlation peak, probably because of less efficient drying of the sample. Clearly no strong correlation signals at ~10 ppm or beyond were observed, indicating the disappearance of hydrogen phosphate units. The small correlation peak at ^1^H/^31^P (~6.3 ppm/~4.1 ppm) could be from an amorphous calcium phosphate phase (ACP) commonly observed in bone mineral.^(^ ^30^ ^)^ Therefore, our results indicated the two populations of MSCs had very different behaviors in the differentiation and mineralization.

The cell sample from the second preparation was then checked again by NMR. The sample had been in an Eppendorf tube for 4 months at room temperature. To our surprise, the 2D ^1^H–^31^P HETCOR experiment, shown in Fig. 4*C* for the cells with 21 days of induction, displayed a very different spectrum compared to Fig. 3*B*, which was obtained on the fresh sample. Figure 4*C* displayed a clear correlation peak at ^1^H/^31^P (~0.0 ppm/~3.8 ppm, the OH^−^/PO_4_^3−^ correlation for HAP). But the originally big correlation peak at ^1^H/^31^P (~9.6 ppm/~3.8 ppm) was significantly attenuated. The peak intensity shifted to ^1^H chemical shift range 5–10 ppm, suggesting ACP phase formation. Therefore, the results indicated that crystalline HAP was formed even for the second preparation after leaving it for a longer period of time at room temperature. Because the viability of the cells would all be lost at this condition, it indicated that the mineral structure could evolve for a period of time after it was released to the ECM.

### Comparison of SSNMR spectra between the bone and the cells

**Fig. 5:**
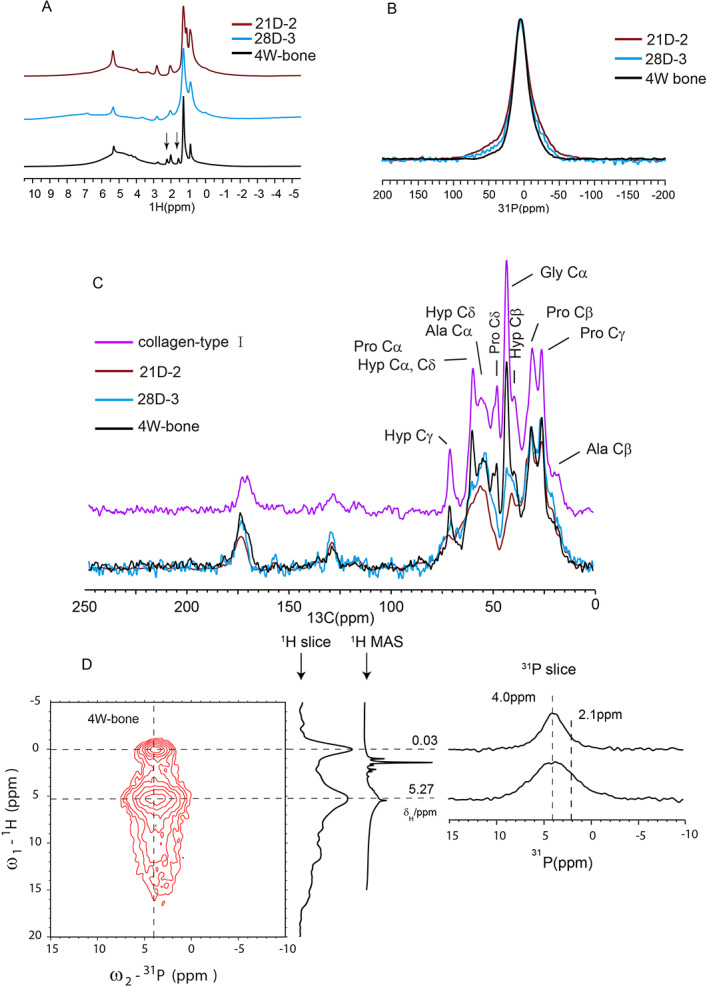
Comparison of SSNMR spectra between osteoblasts from MSCs differentiation and the rat bone. The spectra from the second preparation with osteogenic induction for 21 days was labeled as “21D‐2” and the spectra from the third preparation with osteogenic induction for 28 days was labeled as “28D‐3” (*A*) 1D ^1^H spectra measured at 15 kHz MAS using 4 scans. (*B*) Static ^1^H‐^31^P CP spectra: 2048 scans for “21D‐2,” 1024 scans for “28D‐3,” and 128 scans for the bone sample. (*C*) ^1^H‐^13^C spectra measured at 15 kHz MAS: 30,000 scans for “21D‐2,” 26,360 scans for “28D‐3,” 3000 scans for the bone sample, and 7000 scans for the collagen‐type I sample. (*D*) 2D ^1^H–^31^P HETCOR spectrum of the bone sample at the freeze‐dried condition: 128 scans were collected.

For further comparison, NMR characterizations of the lower limb bone from a 4‐week‐old rat were carried out. The bone sample was ground into powder and lyophilized before the NMR measurement. The 1D ^1^H MAS spectrum of the bone sample (black line in Fig. 5*A*) was similar to the ^1^H spectrum of type‐I collagen in the literature,^(^ ^32^ ^)^ whereas the ^1^H spectrum of the cells showed clear differences (two extra peaks in the spectrum of bone were indicated by the arrows). Similarly, the ^1^H‐^13^C CP MAS spectrum of the bone sample in Fig. 5*C* showed a typical spectrum of collagen with hydroxyproline, proline, and glycine peaks,^(^ ^24^ ^)^ which was very different from the cell samples. The two cell samples also showed differences between themselves. In order to provide a more quantitative description on the similarity and difference of the spectra, we tried to align and normalize the peak at 24.1 ppm and obtained the peak integration centered at 70.5 ppm, 61.4 ppm (the peaks for hydroxyproline [Hyp]), and 40.7 ppm (the peak for glycine). We found all three peaks showed higher intensities for the collagen and bone samples and all three peaks were higher for 28D‐3 than 21D‐2. The root‐mean‐square deviation (RMSD) was also calculated to show the difference (Table S1), with a smaller RMSD indicating a higher similarity to collagen. Our results indicated that the third preparation (28D‐3) had more collagen characteristics in the spectrum, which was consistent with the faster HAP formation for the third cell preparation. The ^1^H and ^13^C spectra therefore indicated the organic matters in osteoblast cells were not dominated by the collagen compared to the bone. The ^31^P CP spectra at the static condition (Fig. 5*B*) were very similar for the three samples, but the bone had a slightly narrower line and the cell sample from the second preparation had the largest CSA width. To further investigate the mineral identity in the bone, the 2D ^1^H–^31^P HETCOR spectrum of bone was obtained. The 2D spectrum in Fig. 5*D* displayed two major correlation peaks at ^1^H/^31^P (~0.0 ppm/~4.0 ppm, the OH^−^/PO_4_^3−^ correlation) and ^1^H/^31^P (~5.3 ppm/~4.0 ppm). The correlation peak at ^1^H/^31^P (~0.0 ppm/~4.0 ppm) indicated HAP was one major ^31^P mineral component in the bone. The ^1^H peak position of the correlation at ^1^H/^31^P (~5.3 ppm/~4.0 ppm) was close to the water peak; however, it displayed the broader linewidth (^1^H from ~3 ppm to ~8 ppm, ^31^P from ~1 ppm to ~8 ppm shown in 1D slices) and a stronger intensity than the surface bound water correlation peak, thereby consistent with the ACP phase. There was a trace amount of water judged from the 1D ^1^H spectrum. From the peak intensity comparison, the relative abundance of the ACP phase was slightly more than that of the HAP phase.

**Fig. 6:**
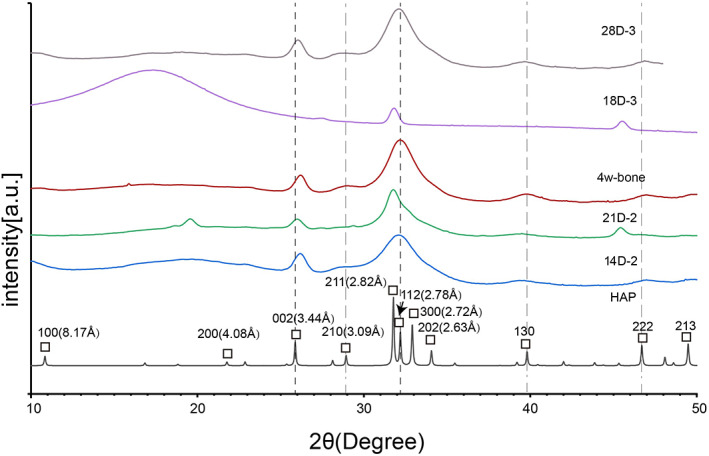
Comparison of the X‐ray diffractogram of MSCs and the rat bone. All samples were freeze‐dried. MSCs from the third preparation with osteogenic induction for 18 or 28 days were labeled as “18D‐3” or “28D‐3.” Cells from the second preparation with osteogenic induction for 14 or 21 days were labeled as “14D‐2” or “21D‐2,” which were freeze‐dried first and allowed to stand at room temperature for more than 4 months before testing. Freeze‐dried bone was also stored at room temperature before testing (labeled as “4w‐bone”). XRD of hydroxyapatite crystals was downloaded from the Inorganic Crystal Structure Database (HAP‐169498‐ICSD); shown in black (labeled as “HAP”).

In order to confirm our finding that HAP was indeed formed by osteoblast cells induced from MSCs, an X‐ray diffraction (XRD) study was also carried out on the cell samples and the bone (Fig. 6). The XRD graph of MSCs after 28 days of induction (labeled as 28D‐3) was exactly the same as that of bone. The diffraction pattern also matched with that of HAP crystal, especially at the 002, 210, 211, 112, 300, 202, 130, and 222 position. Both cells and the bone showed XRD with broader peaks and less resolutions compared to that of the HAP crystal, indicating inhomogeneous nature. The XRD results indicated HAP formation for the second preparation too after 4 months of incubation, confirming the NMR result. The difference in the XRD could also be seen for cell samples with different induction time, indicating the mineral structural evolution. TEM images were also obtained for the second preparation, taking the sample out of the rotors and the third preparation. The images showed fibril‐like structures and dark mineral bands consistent with the highly mineralized collagen fibrils (Fig. S5).

## Discussion

To accurately know the cellular status and the cell mineralization would be very important in bioengineering. The MSC is a highly heterogeneous population with different differentiation potentials. No molecular markers or marker combinations could be used to gain a reliable characterization of the stemness of MSC population or to judge the purity of MSC culture.^(^ ^33^ ^)^ In this study, three preparations of the cells displayed different behaviors observed by SSNMR. The first preparation showed the least mineral deposition. MSCs for this preparation was obtained from a 3‐month‐old rat and the Asc concentration used was relatively low (50μM). But the second and the third preparation were the same, using cells from younger rats with the same age (2 weeks old) and the Asc concentration was higher (400μM). The microscopic images could not show a difference helping us judge the mineralization process. The MSCs differentiation and the osteoblast mineralization were displayed with clear markers in NMR spectra. Therefore NMR could be used in drug screening or quality control of the cells.

In our study, the cells and ECM were not separated or disturbed by harsh chemicals. This provided us the opportunity to observe the structural changes of minerals in ECM. For the second preparation, the less efficient mineral conversion allowed us to observe the mineral intermediates before they were converted to HAP and ACP. The mineral intermediate contained HPO_4_^2−^, suggesting dicalcium phosphate dihydrate as an early phase of the mineral deposit reported in the literature.^(^ ^34^ ^)^ This observation confirms that the bone mineralization can be considered as two stages: the early stage of a cellular process and the later stage of a chemical process of calcium phosphate transformation and HAP crystallization, mainly achieved in the ECM. The later stage could be facilitated by the proteins and the chemicals in the ECM. That more collagen in the cell sample for the third preparation is consistent with its fast mineralization ability. This is common to the other biomineralization processes; eg, the enamel formation is believed to be facilitated by the protein matrix without the cells.^(^ ^23^ ^)^ Because the bone mineral is a combination of HAP and ACP, it suggests that a full conversion of phosphorus minerals to HAP may not be necessary to be desired.

Finally, the advantage of NMR in studying the cells as a whole‐cell state could be explored further with the help of the advanced NMR pulse schemes and signal enhanced techniques, such as dynamic nuclear polarization (DNP). It would be interesting to study the protein and mineral interactions using the whole‐cell sample or study the interactions between different types of the cell because the bone biomineralization involves many types of the cells.

In conclusion, our studies for the first time, utilizing SSNMR, displayed the whole process from the MSC differentiation into osteoblast to cell mineralization at the molecular level. Our studies reveal SSNMR is quite sensitive to biomineral‐associated signaling in this cellular system. It is well known that the biomineralization process is complex, and deviation from normal biomineralization process will lead to a variety of diseases, such as osteomalacia, disorders of extraskeletal mineralization, rickets, osteoporosis, and osteonecrosis of mandible (caused by adverse effect of bisphosphonates, which is the first choice of drug to treat osteoporosis).^(^ ^35^ ^)^ Lack of detailed molecular level and cellular level knowledge on the cause make it difficult to develop effective drugs for treating biomineral‐associated diseases. SSNMR is a powerful tool to provide this detailed knowledge, and give us the way to overcome biomineral‐associated diseases.

## Author Contributions

**Jing‐Yu Lin:** Investigation; writing – original draft; writing – review and editing. **Ming‐Hui Sun:** Investigation; writing – original draft. **Jing Zhang:** Investigation; methodology. **Meng Hu:** Investigation. **Yu‐Teng Zeng:** Investigation; methodology. **Qian‐qian Yi:** Investigation. **Jian Wang:** Methodology. **Yun Bai:** Conceptualization; methodology. **Yifeng Zhang:** Conceptualization; funding acquisition; investigation; supervision; writing – review and editing. **Junxia Lu:** Conceptualization; formal analysis; funding acquisition; supervision; writing – original draft; writing – review and editing.

## Conflicts of Interest

The authors declare no conflict of interest.

### Peer Review

The peer review history for this article is available at https://publons.com/publon/10.1002/jbm4.10662.

## Supporting information


**Appendix S1** Supporting Information
Figs. S1–S5
Table S1

## Data Availability

All NMR data and experimental results are available upon request from the corresponding authors.
