# Supplementary material for: Solid‐State Nuclear Magnetic Resonance (SSNMR) Characterization of Osteoblasts From Mesenchymal Stromal Cell Differentiation to Osteoblast Mineralization
Source: JBMR Plus. 2022 Sep 12;6(10):e10662. doi: 10.1002/jbm4.10662 (PMC9549719; doi:10.1002/jbm4.10662)
Supplement: Supplementary file 1 — Appendix S1 Supporting Information Figs. S1–S5 Table S1 [file JBM4-6-e10662-s001.docx]

**Solid-state NMR (SSNMR) Characterization of Osteoblast from Mesenchymal Stromal Cell Differentiation to Osteoblast Mineralization**

Jing-Yu Lin^+[a,b]^, Ming-Hui Sun^+[a,b]^, Jing Zhang^[a,b]^, Meng Hu^[a,b]^, Yu-Teng Zeng^[a,b]^, Qian-Qian Yi^[a,b]^, Jian Wang^[a]^, Yun Bai^[a]^ Yifeng Zhang* ^[a,c]^ and Jun-Xia Lu*^[a]^

[a] School of Life Science and Technology, ShanghaiTech University, Shanghai, 201210, China

[b] University of Chinese Academy of Sciences, Beijing 100049, China

[c] Shanghai Clinical Research and Trial Center, ShanghaiTech University, Shanghai, 201210, China

+ contributed equally

* Corresponding author: Jun-Xia Lu, phone:86-21-20684538, fax: 86-21-20685052, E-mail: [lujx@shanghaitech.edu.cn](mailto:lujx@shanghaitech.edu.cn); Yifeng Zhang E-mail: zhangyf3@shanghaitech.edu.cn

Figure S1. The deconvolution of static ^1^H-^31^P CP spectra of the whole cell samples using DMFIT^(1)^, in which MSCs were osteogenically induced for 14 days by 50 μM ascorbic acid or 400 μM ascorbic acid in combination with 200 nM Dex and 20mM β-GPO_4_. Three simulated components (the red, blue and green lines) were used with one as an isotropic component (green). The experimental CP spectra were shown in purple and the summation of the three components was shown in black. The contribution of each component was summarized in the table below the figure with δ_CS_=σ_33_-σ_iso_, η_CS_=(σ_22_-σ_11_)/δ_CS_.


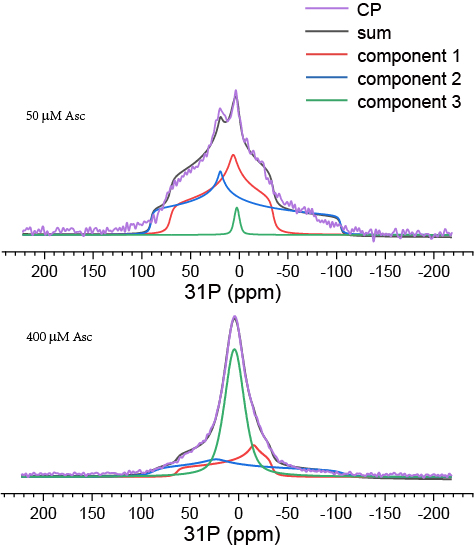


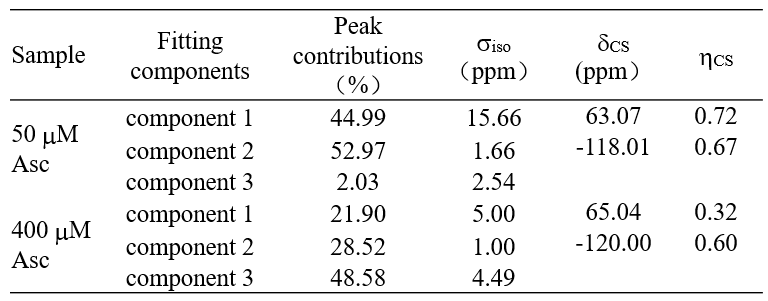


Figure S2. The deconvolution of ^1^H-^31^P CP MAS spectra of the whole cell samples at 15 kHz MAS using DMFIT, in which MSCs were osteogenically induced for a period of 21 days by 400 μM ascorbic acid in combination with 200 nM Dex and 20mM β-GPO_4_. Two components (the red and blue lines) were used. The experimental CP spectra were shown in green and the summation of the two components was shown in black. The contribution of each component was summarized in the table below the figure with δ_CS_=σ_33_-σ_iso_, η_CS_=(σ_22_-σ_11_)/δ_CS_.
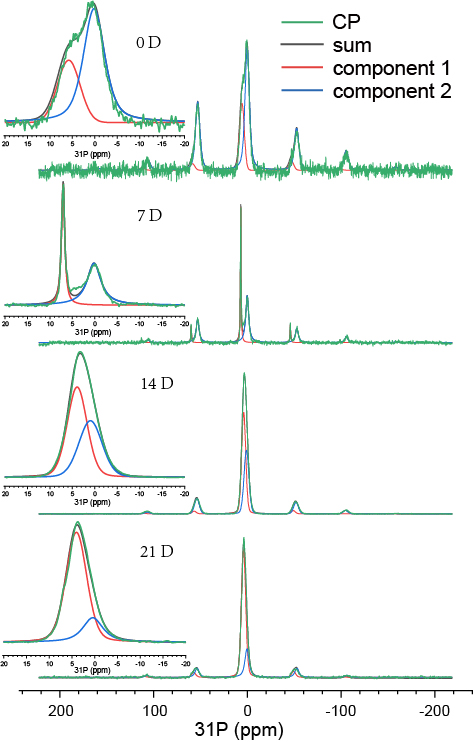


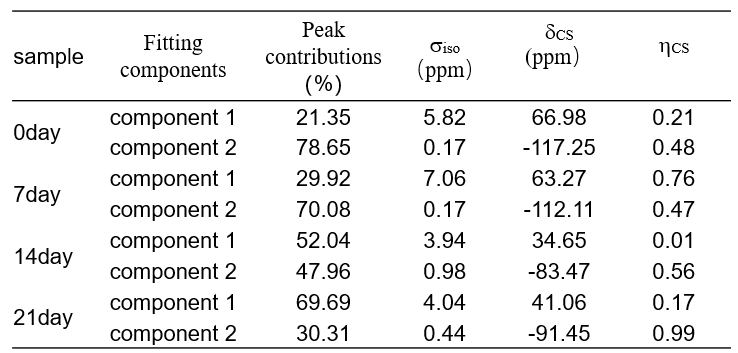


Figure S3. Optical microscopic observation of osteogenically induced cell samples without staining. (A) was for the 2nd preparation after 21 days’ induction. (B) was for the 3rd preparation after 28 days’ induction. The yellow squares were expanded twice to show a better view.


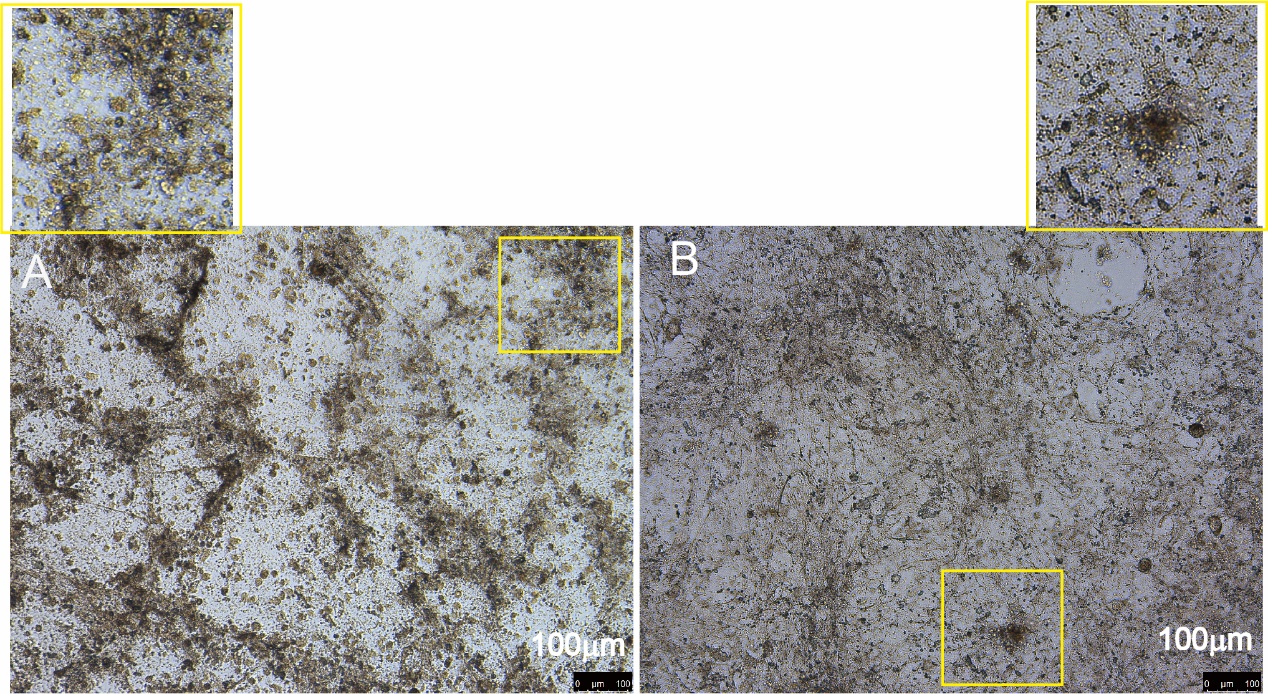


Figure S4. The deconvolution of ^1^H-^31^P CP MAS spectra of the whole cell samples at 15 kHz MAS using DMFIT, in which MSCs were osteogenically induced (the 3^rd^ preparation) for 18 days or 28 days by 400 μM ascorbic acid in combination with 200 nM Dex and 20mM β-GPO_4_. Two-components deconvolution was used for the spectrum of cells with 18 days’ induction, but three-components deconvolution was used for the spectrum of cells with 28 days’ induction. The experimental CP spectra were shown in black and the summation of the all components was shown in red. The contribution of each component was summarized in the table below the figure with δ_CS_=σ_33_-σ_iso_, η_CS_=(σ_22_-σ_11_)/δ_CS_.


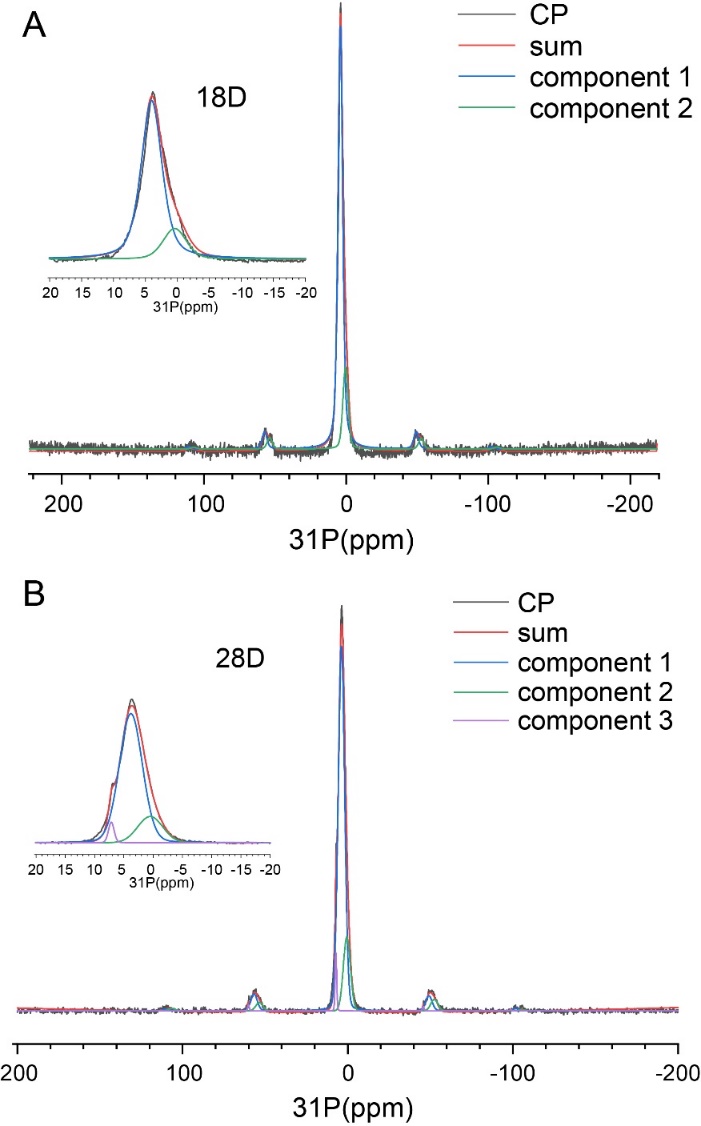


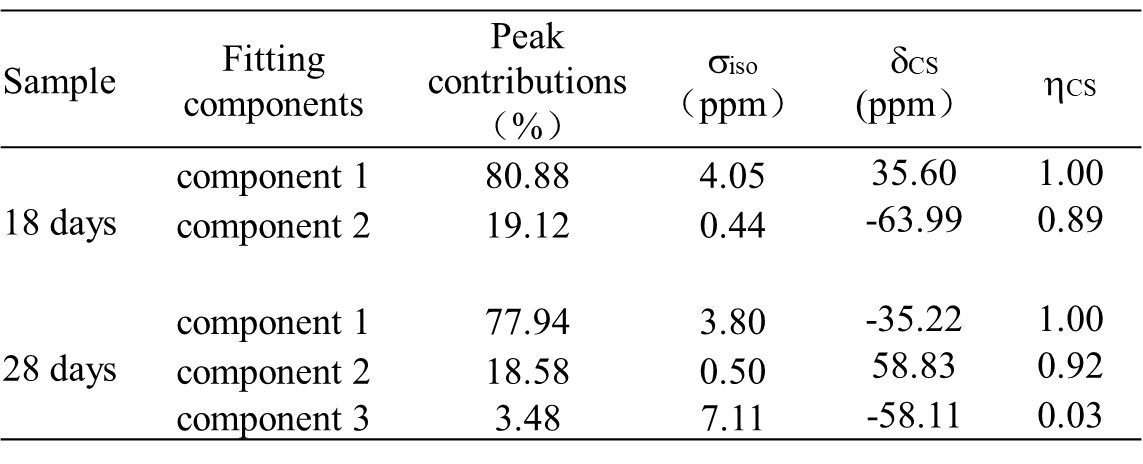


Figure S5. The TEM images of the cell samples after osteogenic induction for 21 days in the second preparation, (A) and for 28 days in the third preparation, (B). For A, the sample was left at the room temperature for 4 months before the imaging. The pictures on the right were the enlargement highlighted in the figure A, B.


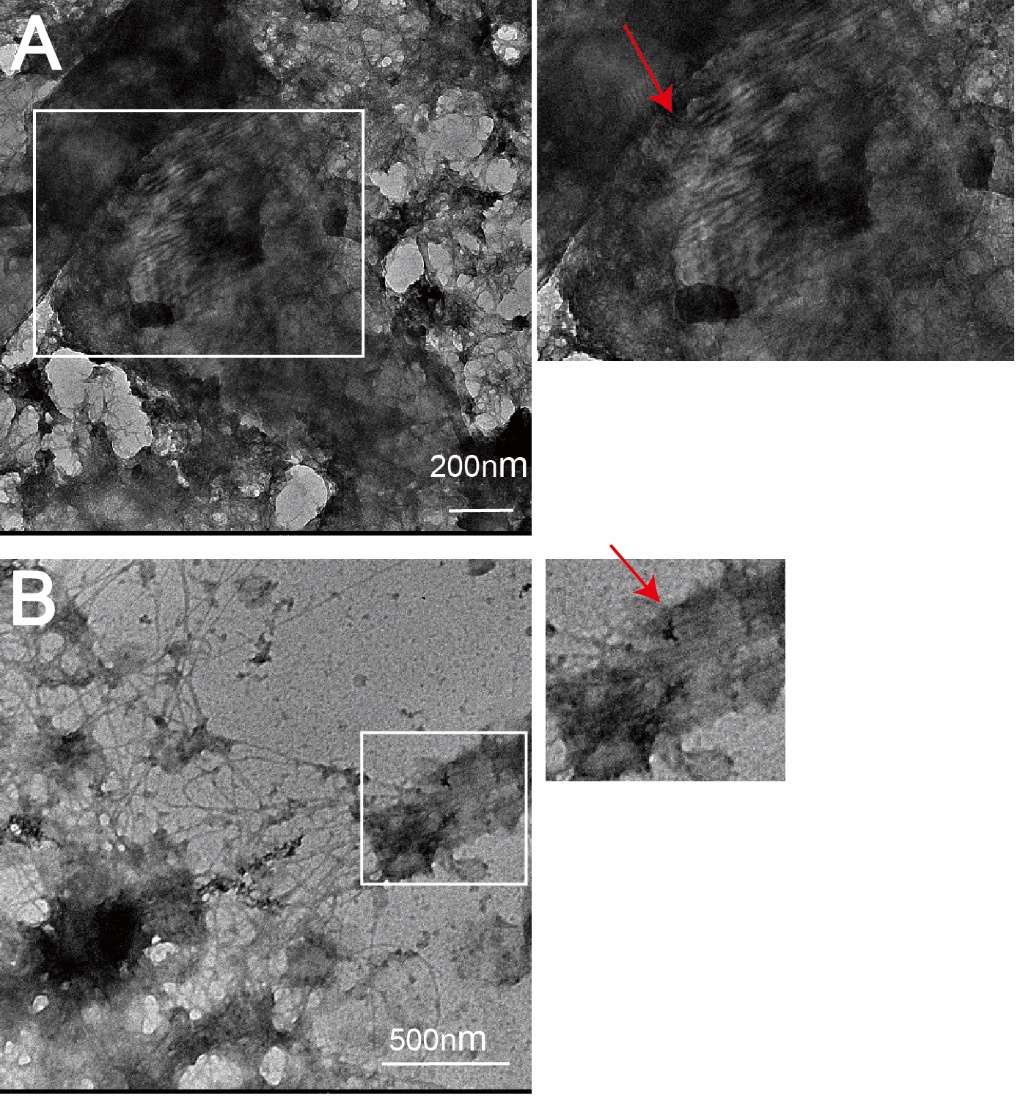


Table S1. The peak integrations (I) for ^1^H-^13^C CP spectra shown in figure 5C. To compare the spectra to that of pure collagen and to quantify the difference, the RMSD values were used, indicating the bone sample the most similar to the collagen. For the cell samples, the third preparation, 28D-3 showed more “collagen like” spectrum then 21D-2. RMSD was defined as[( (ΔI_70.5_)^2+(ΔI_61.4_)^2+(ΔI_40.7_)^2)/3]^0.5.

| Peaks (ppm) (integration range) | collagen | 4w-bone | 28D-3 | 21D-2 |
| --- | --- | --- | --- | --- |
| 70.5 (69.0-72.0) | 0.21 | 0.23 | 0.19 | 0.12 |
| 61.4 (57.1-65.8) | 0.94 | 1.09 | 0.92 | 0.78 |
| 40.7 (35.4-45.9) | 2.07 | 1.81 | 1.29 | 0.88 |
| 24.1 (20.7-27.4) | 1.00 | 1.00 | 1.00 | 1.00 |
| RMSD |  | 0.17 | 0.45 | 0.70 |

Reference

1. Massiot, D.; Fayon, F.; Capron, M.; King, I.; Le Calve, S.; Alonso, B.; Durand, J. O.; Bujoli, B.; Gan, Z. H.; Hoatson, G., Modelling one- and two-dimensional solid-state NMR spectra. *Magn Reson Chem* **2002,** 40, (1), 70-76.
